# Factors affecting tobacco smoking in Ethiopia: evidence from the demographic and health surveys

**DOI:** 10.1186/s12889-019-7200-8

**Published:** 2019-07-12

**Authors:** Harminder Guliani, Samuel Gamtessa, Monika Çule

**Affiliations:** Department of Economics, University of Regina, 3737 Wascana Parkway, Regina, SK Canada

**Keywords:** Tobacco smoking, Khat chewing, Two-stage residual inclusion, Multi-level analysis, Ethiopia

## Abstract

**Background:**

Tobacco smoking is growing at an alarming rate in the developing world and sub-Saharan Africa. Although Ethiopia has a relatively low rate in the region, it is not immune to the tobacco epidemic. The government of Ethiopia passed an anti-tobacco bill in 2015 that includes measures governing tobacco consumption, advertising, packaging, and labeling. To effectively address the challenge of tobacco control, the government should consider a number of aspects of tobacco production and consumption, such as local production in rural areas, as well as the complementarity nature of tobacco and khat use.

**Methods:**

Using the World Bank’s Demographic and Health Surveys (2011 and 2016), this paper analyzes the key determinants of tobacco smoking in Ethiopia, emphasizing possible differences in various social contexts, across regions. More specifically, we assess the association between khat use and tobacco smoking while controlling for various observed individual-level, household-level, and community-level covariates. Using GPS data, we are able to capture the neighboring effects of smoking behavior in community clusters bordering other administrative regions as well as differences in smoking patterns between lowland and highland residents. We utilize a multilevel modeling framework and use a two-stage residual inclusion estimation method that accounts for the endogeneity of khat and tobacco use.

**Results:**

The results suggest that chewing khat and geographic regions are statistically significant determinants of tobacco smoking even after controlling for various socioeconomic and demographic factors. Altitude information analysis suggests that people living in lowlands are more likely to smoke compared to those living in highland areas. Additional analysis including interactions between regions and khat use indicate wide inter-regional variations in tobacco smoking by khat users. We also extend our analysis by interacting khat use with religious adherence. Results indicate a wide variation in tobacco smoking by khat chewers across different religious groups.

**Conclusions:**

To effectively control tobacco smoking of the diverse communities in Ethiopia, policymakers should consider a multi-pronged policy approach that combines various policy tools that account for regional variation, the local social contexts, as well as the complementary nature of smoking and khat chewing practices.

**Electronic supplementary material:**

The online version of this article (10.1186/s12889-019-7200-8) contains supplementary material, which is available to authorized users.

## Background

Despite decades of tobacco control policies, smoking kills more than seven million people each year. It is estimated that over 86% of these deaths are from direct tobacco use, while around 13% are due to secondhand smoke [1]. The low and middle-income countries are especially facing an increasing burden of tobacco related diseases. Africa, particularly sub-Saharan Africa, is experiencing increasing tobacco use. The prevalence of smoking varies considerably among African countries, ranging from 4% in Ghana to 27.2% in Lesotho [2, 3]. Some countries have seen a rapid and significant increase in consumption over the past 16 years. More specifically, Mozambique and Nigeria have seen 220 and 60% growth in cigarette consumption, respectively [4]. Rising population growth, increased consumer purchasing power due to economic growth, and lax enforcement of tobacco control policies are among the contributing factors that make the African region more susceptible to the tobacco industry [4].

Ethiopia is one of the sub-Saharan countries that have relatively low rates of tobacco smoking in the region. Nevertheless, it is not immune to the tobacco epidemic. According to the World Health Organization (WHO) (2015), 8.9% of Ethiopian men and 0.5% of Ethiopian women age 15 years and older smoke tobacco products [5]. Every year, more than 16,800 Ethiopians are killed by tobacco-related diseases such as tuberculosis, lung cancer, cardiovascular and respiratory disorders. In addition, the direct and indirect cost of smoking in Ethiopia is considerable and it is estimated to be 1391 million Birr (about USD 50 million) [6]. In an attempt to control tobacco consumption, the government of Ethiopia became a party to the WHO framework convention on tobacco control on June 23, 2014, and then followed with passing the anti-tobacco bill in 2015.^1^ These regulations, based on Tobacco Control Directive (no. 28/2015), include measures governing consumption, tobacco advertising, packaging and labeling, and other various product regulations [7].

While these regulations are welcomed first steps in controlling tobacco consumption, it is important to recognize that their effectiveness is blunted, in the Ethiopian context, due to rural-urban differences in smoking practices, the existence of the unregulated/illicit tobacco economy and the income related market segmentation [8]. For instance, in the rural areas locally produced tobacco products (e.g water pipes locally known as gaya) are sold and consumed in open markets. These products are not subject to taxation or other types of modern regulatory requirements with respect to labeling or advertising. In other words, the tobacco control regulations have no enforceability in the unregulated/illicit tobacco economy and as a result, they have little to no effect on the underlying behavior of tobacco consumers in those markets.

In addition, income disparity has resulted in a clear segmentation of tobacco retail markets in urban centers. While local producers such as the Ethiopian National Tobacco Enterprise cater to low-income smokers with cheaper and lower quality brands (Gissila and Nyala among others), the imported and relatively more expensive brands are only consumed by high-income urban residents. This market segmentation within the urban centers, as well as the rural-urban differential smoking practices, suggests that policies that utilize consumption taxation as a means to moderate the prevalence of smoking, or banning smoking in certain areas, have differential effects in reducing smoking and the external harms of secondhand smoke. To effectively control tobacco consumption in Ethiopia, it is important that policymakers design policies that account for risk factors affecting tobacco consumption within their proper contexts.

### Tobacco smoking and khat

Another consideration with relevance of tobacco consumption in the Ethiopian context is the rising consumption and production of khat (*Catha edulis*). Khat is a plant leaf that has been used as a stimulant for recreational purposes for centuries in the Horn of Africa and the Arabian Peninsula [9]. In terms of production, khat has become a large export crop over the past two decades, following coffee and oilseed. Khat trade is booming, with many growers switching production from coffee to khat [10].^2^

In terms of consumption, currently, 16% of the nation’s 100 million population is chewing khat, with wide variation in consumption across the country. For instance, while in the Harari region in eastern Ethiopia the prevalence rate is 53%, in the Tigray region in northern Ethiopia this rate is only 1.1% [11]. The DHS data (from both 2011 and 2016 survey years) used in this study suggests that about 17% of Ethiopian respondents reported chewing khat in 30 days preceding the survey. In addition, a meta-analysis on the prevalence of khat chewing among Ethiopian university students indicated that more than one in five students have been engaged in khat chewing [12]. Furthermore, it appears that there is an increase in local consumption. Before the beginning of the twenty-first century only limited amount of khat was chewed for socialization and at religious gatherings among Muslims, while currently, the number of khat chewers has increased among all socio-demographic characters [13]. The Economist (2017) also reports that khat kiosks are spotted around all main towns along with young men chewing on street corners or in university libraries [10].

Increasing khat consumption has become a growing public health concern. Potentially due to its WHO status as a drug of abuse [14], khat has been banned in the US, Canada, Uganda, and some European countries. Evidence suggests that regular khat consumption causes psychological dependence and can have serious health consequences including increased blood pressure, cardiac abnormalities, higher rates of mental disorder, dental health problems, and gastrointestinal disorders [15–19]. In addition, khat dependence is also associated with economic and societal adverse effects such as higher household expenditures on khat consumption, strained family relationships and anti-social behavior [20]. It should be noted that while the production of khat is legal in Ethiopia, chewing khat is unregulated despite its well-known negative consequences [11, 21].

One reason that we pay attention to khat consumption is that in the African region, khat is often consumed concurrently with tobacco. A recent review by Kassim et al. (2015) on the epidemiology of tobacco use among khat users suggests that khat chewers often smoke tobacco concurrently with khat in order to increase its effects [22]. Nakajima et al. (2016) analyzed the concurrent use of khat and tobacco in Yemen [23]. They reported that 70% of concurrent users started (or were exposed to) khat chewing prior to cigarette smoking. They also found that earlier exposure to khat use is associated with earlier consumption of tobacco. They also found an elevated volume of consumption - a greater number of cigarettes smoked – occurred while chewing khat.

The practice of concurrent use of khat and tobacco is present in Ethiopia as well. A study by Kebede (2002) reports that 20% of survey respondents, had started smoking cigarettes and chewing khat at the same time [24]. Another WHO study on chronic disease risk factor surveillance also reports that 15.4% of current khat user adults in Ethiopia smoke while chewing khat [25].

Given the well-known public health benefits of reducing tobacco consumption, our study aims to understand the main determinants of smoking in Ethiopia, giving khat consumption a particular consideration. Section 1.2 that follows provides a summary of the various studies on the determinants of tobacco smoking in Ethiopia. The section also outlines the gaps in the literature and the contribution of our study to fill these gaps.

### Tobacco smoking in Ethiopia: what do we know?

The existing literature on the determinants of tobacco smoking in Ethiopia is fairly limited. Sreeramareddy et al. (2014) analyzed the prevalence, distribution and social determinants of tobacco use in the sub-Saharan African region, using national-level data for 30 countries, including Ethiopia [26]. Since their findings relied on the pooled data, with Ethiopia being one of the observations, it is difficult to infer the significance of their findings for the case of Ethiopia. Other studies with a specific focus on Ethiopian contexts (for instance, a given schools/university setting) and localities (a particular town or city) utilize fairly small samples. Dereje et al. 2014, Eticha and Kidane 2014, Reda et al. 2012, Rudatsikira et al. 2007, Schoenmaker 2005, studied determinants of cigarette smoking among adolescents and school-age students in different parts of Ethiopia [27–31]. They found that sex (male), older age (older youth had a larger tendency to smoke), parent’s smoking habit, alcohol use, perception about the health risks of smoking cigarettes, religion (Islam), and smoker friends are statistically significant predictors of tobacco smoking.

Similar results are reported in Reda et al. (2013) which considers 548 individuals age 15 and above in a rural town and its rural surroundings in eastern Ethiopia [32]. While these studies provide useful insights into the determinants of smoking, they focus on a subset of the population and are not national in scope and therefore, their findings cannot be generalized to all jurisdictions. Ethiopia is subdivided into nine administrative regions and two chartered cities that are very different in sizes, demography, lifestyle, and culture. The regional variation is thus expected to affect the prevalence of smoking. Using the 2011 DHS data at the national level and logistic regression model, Lakew and Haile (2015) are the only ones to consider regional variations in their analysis of the determinants of tobacco use [33]. They found significant regional variations in the prevalence of tobacco use. In addition, Haile and Lakew (2015) is the first study to analyze the factors associated with khat chewing practices among Ethiopian adults [11], even though they did not analyze the association between khat chewing practices and smoking behavior.

Kebede (2002) analyzed the current and lifetime prevalence of cigarette smoking and khat chewing among 1103 university and college students in North West Ethiopia [24]. As noted earlier, about 20% of survey respondents started chewing khat and smoking cigarettes at the same time, suggesting strong complementarity between the two practices. Although Kebede (2002) is an exception in discussing the possible correlation between smoking tobacco and chewing khat [24], the relation between these practices and whether khat chewing is an important factor in the likelihood of tobacco smoking, is not examined empirically.

Our study aims to fill the gaps in the existing literature through a robust and rigorous empirical examination of various determinants of tobacco smoking in Ethiopia. By using the 2011 and 2016 Ethiopian Demographic and Health Survey we ensure inclusion of regional variation in our analysis. Furthermore, a novel contribution of our study, which enriches the analysis of geographical variation, is the utilization of GPS data in two different ways. First, we capture the spillover effects of neighboring communities by looking at similar smoking patterns between the community clusters bordering other administrative regions. Second, we look into differences in tobacco smoking between highland and lowland areas.

In addition to understanding the geographic variation of tobacco smoking, we pay specific attention to the impact of khat chewing practices and other factors affecting smoking in Ethiopia, at the individual level, the household level, and the community level. Unlike Lakew and Haile (2015) that utilize the same data (2011 DHS) [33], we use multilevel modeling, which allows us to control for some unobserved community-level factors that could influence tobacco smoking within a community. To account for the endogeneity of khat chewing practices as well as the possible problems related to omitted variables, we utilize a two-stage residual inclusion method. Our analysis also employs interaction variables, to better understand whether there are any inter-regional and religion-based differences in khat chewing practices and tobacco smoking. Finally, the use of 2016 Survey data, in addition to the 2011 data, expands the sample size considerably and allows us to observe any possible differences in tobacco smoking at different times. Such rigorous analysis of various determinants of smoking would provide policymakers with valuable insights to design policy interventions that are properly suited to each social context and jurisdiction.

## Methods

### Data

The analysis uses the 2011 and 2016 DHS data for Ethiopia collected by the Ethiopia Central Statistical Agency (CSA) with technical assistance from ICF International and funding through the United States Agency for International Development (USAID). The DHS is a large-scale cross-sectional household survey that uses a multistage cluster sample design to collect information on nationally representative samples of males and females. The overall sample in this study consists of 56,644 adult respondents between the ages of 15–49 years,^3^ of which 32,198 are female and 24,446 are male. The 2011 data consists of 29,383 adult respondents of which 16,515 are female and 12,868 are male. The 2016 data consists of 27,261 adult respondents of which 15,683 are female and 11,578 are male.

The survey follows an international methodological approach and is conducted every five years. The Ethiopian DHS samples were selected using a stratified, two-stage cluster sampling design.^4^ DHS collects, among other things, information on the use of various types of smoking and chewing tobacco, as well as data on respondents’ various socio-economic and demographic characteristics such as age, education, religion, wealth index, place of residence, administrative regions, and employment status.

The DHS also collects GPS data, including latitude and longitude of the center of the sample cluster, which can be linked to household-level and individual-level attributes contained in the full DHS dataset. GPS dataset is used in our analysis to calculate two distance variables: first, the Euclidean distance between the high smoking regions and community clusters from the neighboring regions to capture their similarity in smoking patterns; second, the altitude information is used to account for differences in tobacco smoking between highland and lowland areas. Anecdotal evidence indicates that lowlanders in Ethiopia are more likely to grow tobacco in their gardens and hence are more likely to smoke, particularly gaya (the local water pipe used to smoke homemade tobacco products).

### Methodology

An individual’s decision to smoke tobacco can, in part, be influenced by unobserved characteristics of the community such as peer influences, the perceived role of tobacco use in facilitating social connectedness, smoking culture and norms, use of other tobacco products, tolerance of tobacco use, and health risk awareness in the community. Therefore, the probability of smoking tobacco is likely to be correlated among community members. This leads to biases in the application of standard logistic regression models [36]. To avoid that, we use a two-level (individual–household and community) random intercept logistic model.^5^

While khat consumers are more likely to smoke tobacco, causation may also operate in the reverse direction. Moreover, some unobserved characteristics may influence both khat chewing practices and tobacco smoking. This may make khat consumption endogenous and produces an upward bias in the estimates of khat. Terza et al. (2008) recommends using two-stage residual inclusion (2SRI) estimation method to address endogeneity in empirical research in health economics and health services [37]. The 2SRI method produces consistent estimates of the parameters involving nonlinear models with endogenous regressors [37]. Therefore, in addition to multilevel modeling, we have used the 2SRI estimation technique to address the endogeneity of regressors and the omitted variables problems. The first stage equation of the 2SRI specifies khat consumption as a function of exogenous variables and the second stage of the 2SRI approach includes the residual computed from first stage estimation as a regressor in addition to other exogenous variables.

To account for the complex sampling design in the DHS, standard weights from the men’s and women’s file were used to adjust for the unequal probability of selection. Per DHS guidelines, these weights were first de-normalized [38]. STATA version 14 **(**StataCorp, College Station, TX) was used for all data analysis.

### Study variables

The outcome variable “current tobacco smoking” is constructed based on the responses provided to the survey questions in men’s and women’s file. The respondents were asked if they currently smoke cigarettes or any other type of tobacco such as cigars, shisha, gaya, hookah during the survey years.^6^ A binary dependent variable was created with a value of zero (0) if the respondent age 15–49 did not smoke any tobacco products and one (1) if the respondent indicated smoking one or more types of tobacco.

Consistent with the existing literature, the independent variables are grouped into three broad categories: individual-level factors, household-level factors, and community-level factors. The individual-level variables include: respondent’s educational attainment (no education, primary education, secondary education, higher-level education); sex (female, male); age (15–19, 20–24, 25–29,30–34, 35–39, 40–49); marital status (married, divorced, unmarried); survey year (2011, 2016); occupation (unemployed, professional, agriculture, unskilled); and current prevalence of chewing khat. The current prevalence of khat chewing is measured by respondent’s khat chewing practices in the 30 days preceding the survey period.

The observed household-level variables include household economic status (measured by wealth index); religion (Orthodox, Catholic, Protestant, Islam, and Traditional); and the household members’ exposure to smoking. The wealth index is calculated by the DHS based on a standard set of household assets, dwelling characteristics, and ownership of consumer items. Each household is classified into quintiles where the first quintile is the poorest 20% of households and the fifth quintile is the wealthiest 20% of households [39]. Household exposure to smoking is a dichotomous variable, which equals one (1) if any household member smokes inside the house and zero (0) otherwise.

The observed community-level variables include the place of residence (urban/rural areas); and the administrative region. The DHS adopts the Ethiopian Central Statistical Agency definition of urban/rural that defines “urban” localities with 2000 or more inhabitants [40]. This definition of “urban” is problematic, as some rural towns are large enough to be categorized as “urban”, despite having a true rural lifestyle and lacking many other basic features one would expect in an “urban” center. As a result, the urban and rural dummy-variables created based on this criterion may provide misleading information and the estimates obtained should be interpreted with some caution.

Administrative regions are represented by eight dummy-variables: Tigray, Affar, Amhara, Oromia, Benishangul-gumuz, SNNP, Gambela, and Eastern.^7^ These community-level variables capture the differences in the availability and accessibility of tobacco products between urban and rural areas and among different regions. While regional dummy-variables could capture differences in smoking patterns across administrative regions, they fail to consider similar smoking patterns for communities that are located close to neighboring regions. For instance, SNNP communities located near the border of Gambela share many social context similarities with communities located in Gambela, which has the highest prevalence of smoking. The regional dummy-variables for the administrative regions, on the other hand, treat these communities as different. Since Gambela has higher smoking prevalence rate than other regions and social context matters for smoking behavior due to imitation, emulation and higher social acceptability toward a widespread practice, we expect the SNNP communities bordering Gambela region to exhibit similar smoking behavior with Gambela communities.

To address this issue, we use DHS’s GPS data to create a distance variable for southern-region (SNNP), community clusters using Gambela as the focal point. Similarly, we created a distance variable for Oromia communities using Harari, the second highest region of smoking prevalence, as the focal point. Although Oromia is a populous, highly-diverse region, its communities that border the Eastern region share many social, religious, and lifestyle characteristics and may behave similarly with respect to smoking.

In addition, for all regions and for each community cluster, we included altitudinal measurements to capture possible differences between lowlands and highlands.^8^ Anecdotal evidence suggests that it is common in lowland areas for households to have small-scale tobacco growing operations, primarily for self-consumption, so including this variable in the analysis will provide some insights on the validity of this anecdotal observation.

Table 1 provides summary statistics for the dependent and independent variables used in the estimation of the likelihood of smoking tobacco.

**Table 1:** Summary Statistics for Dependent and Independent Variables (*N* = 56, 644)

Variables	Mean	S.D
*Dependent Variable*		
Smoking Tobacco	0.04	0.18
*Individual-level independent variables*		
Chewing khat in the 30 days preceding the survey	0.17	0.38
Sex		
Male	0.52	0.50
2016 Survey Year	0.55	0.50
Age		
15–19	0.22	0.41
20–24	0.17	0.37
25–29	0.17	0.38
30–34	0.13	0.33
35–39	0.12	0.32
40–49	0.15	0.36
Marital Status		
Not Married	0.33	0.47
Married	0.61	0.49
Divorced	0.06	0.24
Education		
No Education	0.40	0.49
Primary	0.43	0.49
Secondary	0.11	0.31
Higher	0.07	0.25
Occupation		
Unemployed	0.26	0.44
Professional/Clerical/Sales/Skilled/Services	0.24	0.43
Agriculture	0.47	0.50
Unskilled Manual/Other	0.04	0.18
Household-level independent variables		
Household wealth quintile		
Quintile 1 (Very Poor)	0.17	0.37
Quintile 2	0.18	0.39
Quintile 3	0.19	0.39
Quintile 4	0.21	0.40
Quintile 5 (Very Rich)	0.25	0.44
Household members smoke inside the house	0.12	0.33
Religion		
Orthodox	0.46	0.50
Catholic	0.01	0.09
Protestant	0.22	0.41
Islam	0.30	0.46
Traditional/Other	0.02	0.13
Community-level independent variables		
Place of residence		
Urban	0.22	0.41
Administrative regions		
Tigray	0.32	0.47
Affar	0.01	0.09
Oromia	0.37	0.48
Benishangul-gumuz	0.01	0.10
SNNP	0.20	0.40
Gambela	0.01	0.06
Eastern (Dire dawa, Harari and Somali)	0.03	0.18
Addis Ababa	0.05	0.22
Distance of SNNP Region from Gambela	0.73	2.07
Distance of Oromia from Dire Dawa	1.84	4.74
Altitude distance relative to the sea level	7.56	0.31

## Results

### Descriptive analysis

Table 2 reports tobacco smoking (in percentages) by selected characteristics for the pooled cross-sectional data. These characteristics include not only the behavior driven characteristics, such as the prevalence of khat chewing practices but also other socioeconomic and demographic characteristics.

**Table 2:** Prevalence of Tobacco Smoking by Selected Variables (*N* = 56, 644)

	Weighted (%)	Unweighted (*n*)
Average	3.21	56,621
Chewed khat in the 30 days preceding the survey	13.38	9462
Household wealth quintile		
Very Poor	4.04	12,814
Poor	3.33	7979
Middle quintile	3.23	7791
Rich	2.22	8483
Very Rich	3.21	19,554
Age		
15–19	0.54	12,695
20–24	1.97	10,219
25–29	3.19	10,332
30–34	4.24	7604
35–39	4.86	6823
40–49	6.03	8948
Sex		
Male	5.72	24,441
Female	0.64	32,180
Religion		
Orthodox	1.77	23,870
Catholic	6.07	487
Protestant	1.22	9791
Islam	6.32	21,680
Traditional/Other	8.93	783
Place of residence		
Urban	3.68	18,141
Rural	3.03	38,480
Administrative Regions		
Tigray	0.75	5774
Affar	8.14	3994
Amhara	0.86	7057
Oromia	4.45	7508
Benishangul-gumuz	7.59	4332
SNNP	2.46	6898
Gambela	12.89	3838
Eastern (Dire dawa, Harari and Somali)	11.17	11,288
Addis Ababa	3.97	5932

Overall, the weighted prevalence rate of tobacco smoking was 3.21% (unweighted total adult population is 56,621with a representative population size of 91,497,740). However, this average mask wide variation in tobacco smoking due to differences in khat chewing practices, economic status, religion, age groups, place of residence, and administrative regions. In the pooled cross-sectional data, 13% of those who chewed khat in the 30 days preceding the survey also reported that they currently smoke tobacco. Respondents who belong to the bottom quintile are, on average, 1.3 times more likely to smoke some kind of tobacco than those belonging to the richest quintile. Male respondents are almost nine times more likely to smoke than female respondents. Respondents who follow traditional religions are seven times more likely to smoke tobacco compared to those who practice the Protestant religion. Urban residents are 1.2 times more likely to smoke tobacco than rural residents.^9^ Finally, older adults are 11 times more likely to smoke tobacco than younger age groups.

**Fig. 1:**
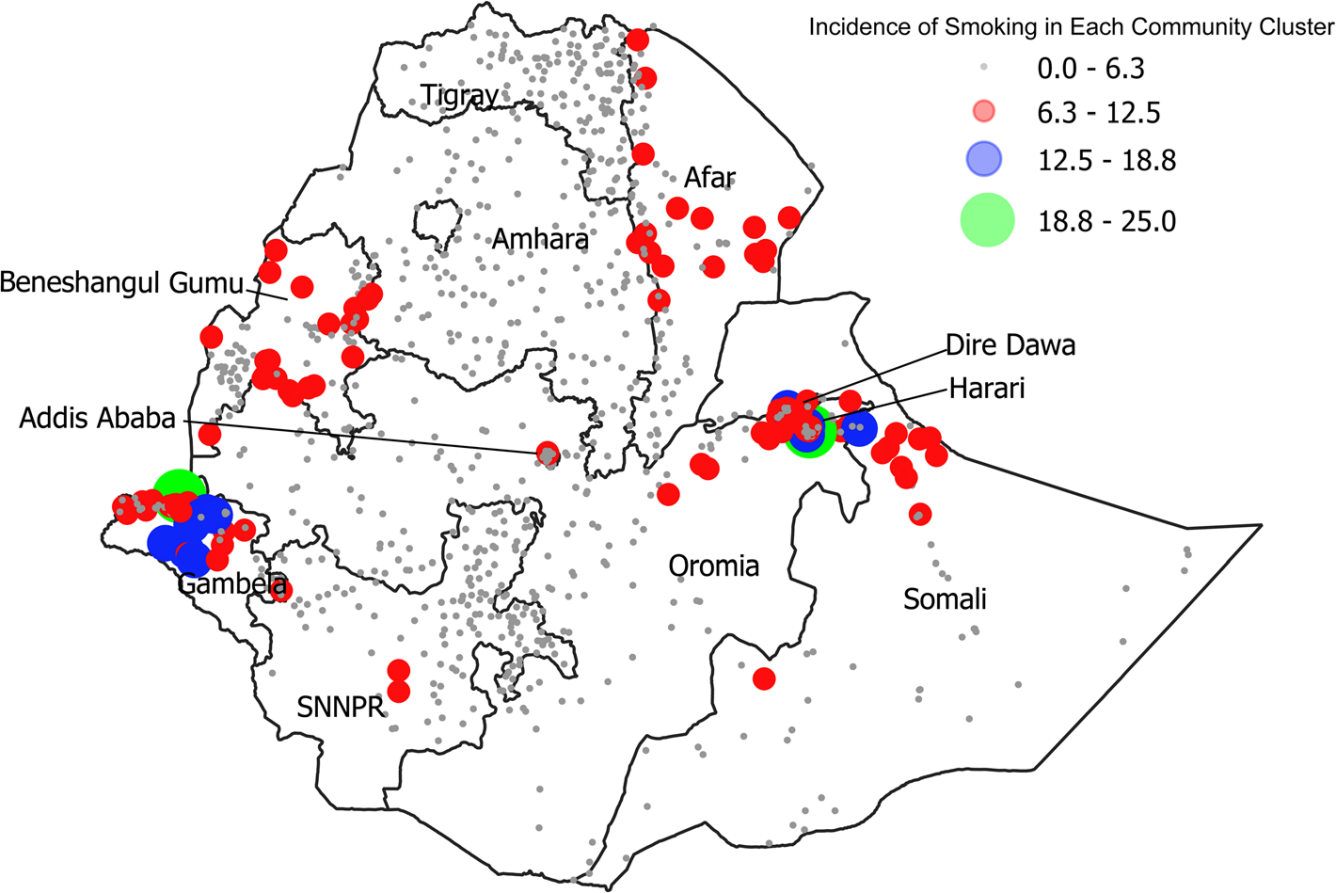
Distribution of Tobacco Smoking by Regions. Illustrates the incidence of smoking in each community cluster, in each region. The size of the bubble corresponds to the prevalence of tobacco smoking within that community. The bigger the bubble, the higher the prevalence of tobacco smoking in that community. Mapped by the authors using the Ethiopian Demographic and Health Surveys (2011 and 2016) and QGIS (V 3.0.2) software. [52]

**Fig. 2:**
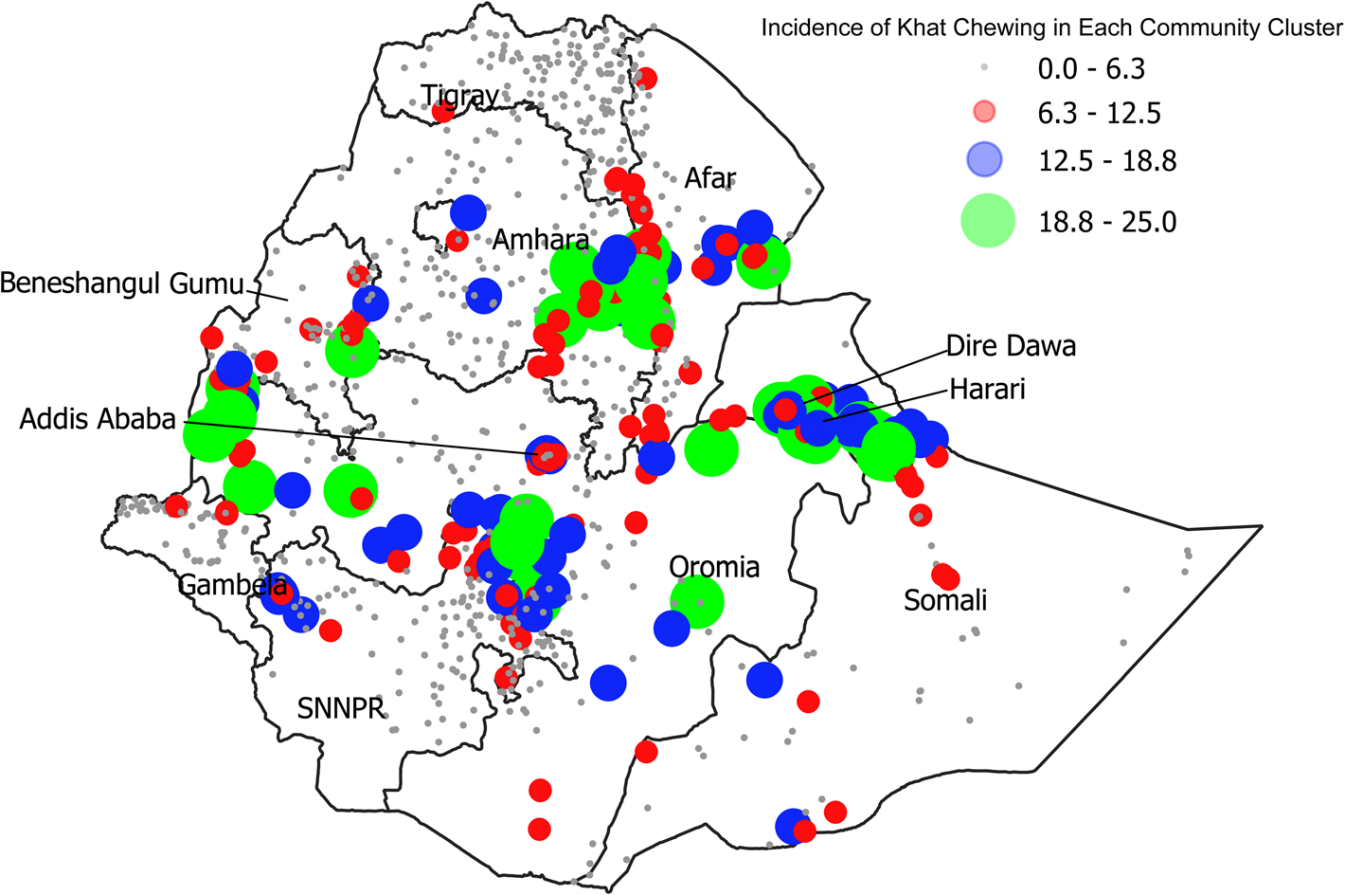
Distribution of Khat Chewing by Regions. Illustrates the incidence of khat chewing in each community cluster, in each region. The size of the community bubble corresponds to the prevalence of khat within that community. The bigger the bubble, the higher the prevalence of khat chewing in that community. Mapped by the authors using the Ethiopian Demographic and Health Surveys (2011 and 2016) and QGIS (V 3.0.2) software. [52]

The prevalence of tobacco smoking in Ethiopia varies widely by the administrative region (Table 2). Gambela residents are 17 times more likely to smoke tobacco than Tigray residents. Figure 1 illustrates the distribution of tobacco smoking across regions in Ethiopia. Each bubble on the map represents the incidence of smoking in each community cluster, in each region. The size of the community bubble corresponds to the prevalence of tobacco smoking within that community. While tobacco-smoking practices are found throughout the country, the highest concentration can be found in the Gambela region, followed by the Eastern region, which includes Dire Dawa, Harari, and Somali. Figure 2 maps the distribution of khat chewing prevalence across Ethiopia. While khat chewing practices are found throughout the country, the highest concentration can be found in the Eastern region, followed by the Amhara region and the Oromia region.

### Econometrics results

The regression results for the likelihood of smoking tobacco are reported in Table 3.^10^ The likelihood-ratio test clearly rejects the null hypothesis that the standard deviation of the random-intercept term is zero and hence favors the random-intercept logistic model over the ordinary logistic model. The intra-community correlation (ρ) and the estimated value of the variance (ψ) of the random-intercept term are also shown in the table. The high value of ρ even after controlling for all observed covariates suggests that there are some unobserved covariates in the primary sampling units that affect an individual’s decision to smoke tobacco. The baseline Model 1 use all explanatory variables including the prevalence of khat chewing.^11^ Motivated by the high regional variation in smoking and Model 1 finding that khat chewers are highly likely to smoke, the regression model is extended by interacting each administrative region with khat chewing practices, to create Model 2. In addition, motivated by the observation in our data that prevalence of khat, as well as the concurrent use of tobacco and khat, vary widely across different religious groups,^12^ we extend the regression by interacting each religious group with khat chewing practices, to create Model 3.

**Table 3:** Regression results for Tobacco Smoking

	Model 1	Model 2	Model 3
Variables	Odds ratio (95% CI)	Odds ratio (95% CI)	Odds ratio (95% CI)
Fixed part			
Individual-level variables			
2016 Survey Year (ref: 2011 year)	0.634* (0.546, 0.737)	0.652* (0.564, 0.753)	0.647* (0.560, 0.747)
Chewing khat in the 30 days preceding the survey	8.162* (6.966, 9.564)	–	–
Sex (Ref: female)	7.065* (5.792, 8.617)	7.536* (6.172, 9.202)	9.908* (8.169, 12.017)
Age (ref: [15–19])			
20–24	3.842* (2.910, 5.074)	3.821* (2.906, 5.025)	4.530* (3.446, 5.955)
25–29	5.951* (4.442, 7.972)	6.007* (4.509, 8.002)	7.437* (5.575, 9.922)
30–34	8.059* (5.923, 10.966)	8.150* (6.026, 11.023)	10.060* (7.418, 13.644)
35–39	8.542* (6.209, 11.750)	8.628* (6.310, 11.797)	10.863* (7.938, 14.865)
40–49	9.579* (7.000, 13.109)	9.640* (7.093, 13.101)	11.909* (8.721, 16.262)
Marital Status (ref: Not married)			
Married	1.078 (0.909, 1.278)	1.078 (0.912, 1.276)	1.144 (0.970, 1.350)
Divorced	1.884* (1.502, 2.363)	1.941* (1.546,2.437)	2.041* (1.635, 2.550)
Education (ref: No Education)			
Primary	1.008 (0.876, 1.160)	1.020 (0.889, 1.172)	1.053 (0.918, 1.209)
Secondary	0.915 (0.748,1.119)	0.936 (0.766,1.143)	0.934 (0.765, 1.140)
Higher	0.566* (0.444, 0.722)	0.596* (0.469, 0.758)	0.568* (0.447, 0.721)
Occupation (ref: Unemployed)			
Professional/Clerical/Sales/Skilled/Services	1.055 (0.864, 1.289)	1.066 (0.871, 1.303)	1.239** (1.014, 1.513)
Agriculture	1.158 (0.945, 1.418)	1.129 (0.922, 1.382)	1.356* (1.108, 1.658)
Unskilled Manual/Other	1.517* (1.131, 2.036)	1.513* (1.130, 2.028)	1.820* (1.360, 2.437)
Household-level variables			
Household wealth quintile (ref: Very Rich)			
Quintile 1 (Very Poor)	2.511* (1.912, 3.297)	2.437* (1.871, 3.174)	2.234* (1.716, 2.909)
Quintile 2	1.804* (1.369, 2.377)	1.780* (1.361, 2.327)	1.683* (1.288, 2.200)
Quintile 3	1.779* (1.330,2.380)	1.746* (1.316,2.317)	1.692* (1.275, 2.246)
Quintile 4	1.170 (0.893, 1.531)	1.172 (0.901, 1.524)	1.150 (0.884, 1.495)
Household members smoke inside the house	11.565* (10.033, 13.331)	11.648* (10.115, 13.413)	12.065* (10.519, 13.838)
Religion (ref: Islam)			
Orthodox	1.226** (1.014, 1.483)	1.027 (0.846, 1.246)	0.300* (0.239, 0.376)
Catholic	2.961* (1.712, 5.122)	2.346* (1.379, 3.990)	1.096 (0.619, 1.940)
Protestant	1.674* (1.268, 2.210)	1.285*** (0.964, 1.712)	0.473* (0.357, 0.628)
Traditional/Other	3.783* (2.439, 5.866)	2.904* (1.882, 4.481)	1.342 (0.831, 2.168)
Community-level variables			
Place of residence (ref: Rural)	2.175* (1.641, 2.884)	2.193* (1.674, 2.872)	2.089* (1.583, 2.756)
Administrative regions (ref: Tigray)			
Addis Ababa	3.638* (2.142, 6.178)	2.353* (1.356, 4.081)	3.537* (2.104, 5.946)
Affar	1.904** (1.089, 3.328)	1.547 (0.864, 2.770)	1.607*** (0.948, 2.726)
Amhara	0.689 (0.399, 1.192)	0.442* (0.230, 0.849)	0.718 (0.418, 1.233)
Oromia	1.563 (0.841, 2.905)	1.700 (0.895, 3.230)	2.281* (1.247, 4.170)
Benishangul-gumuz	4.890* (2.941, 8.131)	4.969* (3.044, 8.110)	3.718* (2.259, 6.121)
SNNP	5.291* (2.186, 12.806)	4.639* (1.947, 11.052)	4.248* (1.770, 10.195)
Eastern (Dire dawa, Harari and Somali)	2.350* (1.450,3.811)	0.649 (0.364, 1.158)	3.070* (1.905, 4.950)
Gambela	7.084* (3.811,13.168)	8.048* (4.438, 14.596)	8.889* (4.846, 16.306)
Distance of SNNP Region from Gambela	0.686* (0.551,0.854)	0.703* (0.561,0.880)	0.731* (0.591, 0.903)
Distance of Oromia Region from Harari	0.967 (0.873, 1.072)	0.948 (0.857,1.050)	0.919*** (0.832, 1.014)
Altitude distance from the sea level	0.593* (0.451, 0.780)	0.602* (0.457, 0.794)	0.735* (0.592, 0.913)
Khat * Region (ref category: Khat Chewing Practices in Tigray)			
Khat * Addis Ababa	–	7.989* (5.354, 11.920)	–
Khat * Affar	–	5.261* (3.716, 7.449)	–
Khat * Amhara	–	10.539* (5.361, 20.719)	–
Khat *Oromia	–	4.836* (3.419, 6.841)	–
Khat * Benishangul-gumuz	–	2.406* (1.543, 3.752)	–
Khat * SNNP	–	5.136* (3.050, 8.649)	–
Khat * Eastern (Dire dawa, Harari and Somali)	–	21.896* (16.109, 29.762)	–
Khat * Gambela	–	1.561** (1.009, 2.413)	–
Khat * Religion (ref category: Islamic khat chewers)			
Khat *Orthodox	–	–	7.072* (5.397, 9.268)
Khat * Catholic	–	–	1.474 (0.591, 3.677)
Khat * Protestant	–	–	6.930* (3.893, 12.339)
Khat * Traditional/Other	–	–	2.028 (0.851, 4.833)
β_ehat_	1.439* (1.093, 1.895)	2.742* (2.038, 3.691)	1.513* (1.102, 2.076)
*Random part*			
ρ^a^	0.17	0.15	0.15
ψ^b^	0.661 (0.526, 0.830)	0.569 (0.449, 0.722)	0.589 (0.459, 0.756)
LR test statistic^c^	356.37*	295.86*	314.22*
Level 1 Units (N)	54,191	54,191	54,191
Level 2 Units	1193	1193	1193

*1% significance level, ** 5% significance level, ***10% Significance level

^a^ Intra-cluster correlation

^b^ Variance of the random-intercept term

^c^ Comparing random-intercept logistic model against ordinary logit model

### Baseline model

The results of Model 1, presented in the second column of Table 3, show that all explanatory variables had the expected signs, and most of them were statistically significant. In line with descriptive statistics, those who chewed khat in the 30 days period preceding the survey are seven times more likely to smoke some type of tobacco. Male respondents are six times more likely to smoke than females. The likelihood of smoking tobacco varied positively by age, with older adults (40–49) being almost nine times more likely to smoke tobacco than the younger age groups (15–19).^13^ Similarly, divorced individuals and those with unskilled manual occupations were more likely to smoke tobacco compared to their counterparts. Compared to those with no education (the reference category), individuals with higher education were 44% less likely to smoke tobacco.

The likelihood of smoking decreased with increasing household wealth. Individuals from the bottom wealth quintile are 1.5 times more likely to smoke tobacco than those from the richest quintile (the reference category). The likelihood of smoking increased (by 11 times) if a household member smokes inside the house.

Compared to those who follow the religion of Islam (the reference category), other religious groups were more likely to smoke tobacco, but to varying degrees. For instance, Catholic followers and traditional followers are more likely to smoke by two and three times, respectively - results that align with the descriptive statistics. In addition, individuals who practice Protestant or Orthodox religions are more like to smoke tobacco than those practicing Islam, by 66 and 22%, respectively. This result is somewhat surprising as the descriptive statistics showed that the prevalence of tobacco smoking among these groups is much lower than among Muslim responders. This result suggests a need for a further investigation which we perform in Model 3. Urban dwellers are more likely to smoke tobacco than rural residents.

We found significant regional variations in tobacco smoking. Specifically, Gambela residents are six times more likely to smoke tobacco than Tigray residents (the reference category), as also suggested by descriptive analysis. Similarly, those who live in SNNP and Benishangul-gumuz are about four times more likely to smoke tobacco compared to Tigray residents. The distance variable further suggests that the residents from the SNNP region that lived closer to the border of the Gambela region, which has the highest incidence of smoking, were 31% more likely to smoke tobacco. Altitude has a statistically significant, negative effect indicating highlanders are 41% less likely to smoke than lowlanders. This finding validates our hypothesis based on the anecdotal evidence. Lastly, the time dummy estimates indicate that the probability of smoking decreased by 37% in 2016 compared to the 2011 survey year.

### Model 2: interaction of region with Khat

Model 2 assesses the inter-regional differences in the probability of smoking by khat users with results shown in the third column of Table 3. The khat prevalence in Tigray region is used as the reference category, a choice motivated by the fact that Tigray has the lowest prevalence of khat chewing practices in the country at 1.1%. The Tigray region is also the only administrative region that has legally banned the production of khat (2009) and has a longstanding ban in consumption [41, 42].^14^ The results of Model 2 show that all estimates maintain the same qualitative effect for the independent variables. Generally, there is negligible change in the odds ratios, but there are a few points worth noting for Model 2 results.

First, Tigray residents (the reference category) who chew khat are the least likely to also smoke tobacco compared to khat users in other regions. Second, although residents who chew khat from all other regions are more likely to smoke tobacco than Tigray residents, the likelihood varied considerably across regions. For instance, individuals who live in the Eastern region and chew khat are almost 21 times more likely to smoke tobacco than Tigray residents. In comparison, khat users that reside in the Amhara region and the capital city Addis Ababa are 10 and seven times, respectively, more likely to smoke tobacco than Tigray residents. Third, the regional difference in an odds ratio of smoking of Eastern residents and Affar residents, when compared to Tigray residents, respectively, become statistically insignificant with the inclusion of the (khat- regional) interaction term. Fourth, the inter-regional differences in khat chewing and tobacco smoking were less pronounced for Gambela residents. Those who chew khat and live in Gambela are about 56% more likely to smoke tobacco than Tigray residents. At the same time, with the inclusion of the regional interaction term, we see that the residents of Gambela are seven times (up from six times in Model 1) more likely to smoke than residents of Tigray. Finally, the inclusion of the regional interaction term with the khat variable results in a slight decrease in the likelihood of smoking when comparing Islam (the reference category) to the other religious groups.

### Model 3: interaction of religion with khat

To assess the differences in the probability of smoking by khat users who practice different religions, the regression model was extended by interacting each religious category with khat use. Model 3 results are presented in the fourth column of Table 3. The khat prevalence among individuals who practice Islam is used as the reference category for the religion-khat interaction term. The result of Model 3 shows that most estimates are robust across all models, confirming the same qualitative effects for most independent variables. While there is negligible change in the odds ratios for most variables, those few that changed expand the story and provide some valuable insights. The following points are worth noting for Model 3 results.

First, overall, individuals from Orthodox and Protestant religious groups become less likely to smoke than the individuals who practice Islam, by 70 and 63%, respectively. Furthermore, the results for Catholics and Traditional religious groups become statistically insignificant. Second, despite the overall lower likelihood to smoke among Orthodox and Protestant individuals, those who chew khat among these groups are six times more likely to smoke tobacco compared to Muslim khat chewers. Third, we noticed that a number of determinants of the prevalence of smoking that lacked statistical significance in Model 1 and 2, gain statistical significance with the inclusion of the (religion-khat use) interaction variable in Model 3. More specifically, in the occupation category, those working in agriculture and professional occupations are more likely to smoke tobacco, than the unemployed (the reference category) by 35 and 24%, respectively.

Finally, unlike the results of previous models where Oromia region and the distance variable (of Oromia community clusters from Harari)^15^ were statistically insignificant, these variables are significant in Model 3. Specifically, Oromia residents are almost 128% more likely to smoke than Tigray residents. The distance variable result shows that Oromia’s bordering communities with the Eastern region are 8% more likely to smoke than those located at farther distances from Harari.

## Discussion

### Baseline model

Our finding that khat users are seven times more likely to smoke some type of tobacco is consistent with Kassim and colleague’s observation [43]. This finding supports the hypothesis that khat use serves as a gateway to tobacco use. Our findings about sex, age, economic status, as measured by the wealth index, and religion are consistent with other studies that show that these factors are statistically significantly associated with tobacco smoking [26, 33].

Significant sex differences in tobacco smoking, with males six times more likely to smoke than females, could be explained by the low social acceptability and cultural norms towards females, where khat chewing and smoking practices are frown upon.

The finding that individuals from the bottom wealth quintile are 1.5 times more likely to smoke tobacco than those from the richest quintile (the reference category) raises concern about the most vulnerable economic groups. Several hypotheses may explain wealth-related inequalities in tobacco smoking. These include, but are not limited to, a lack of awareness of health risks among the poor and their inability to deal with the stress of their economic conditions [44]. In their study of the economic impact of tobacco consumption on the poor in Bangladesh, Efroymson et al. (2001) suggest that poor people may choose to purchase cigarettes over meeting basic needs such as food, clothing, health care, and education, which further aggravates poverty [45].^16^

It is not surprising that we find that the likelihood of smoking increased by 11 times if a household member smokes inside the house. In many rural parts of Ethiopia, while men smoke in both public places and in their homes, women mainly smoke in their homes, which often consist of small and poorly ventilated rooms [46]. The combined DHS data (2011 and 2016 survey) suggests that 52% of female respondents reported to smoke inside the house. One expects that imitation and emulation of behaviors of the family members to take place. Being exposed to smoking on a regular basis would entice other family members to smoke themselves. As a result, more household members are exposed to smoking-related diseases.

The existing studies on Sub-Saharan Africa provide mixed evidence on urban/rural tobacco smoking. While our result that urban dwellers are more likely to smoke tobacco than rural residents, is consistent with Pampel (2008) [47], Sreeramareddy et al. (2014) found that rural residents are more likely to smoke than urban residents [26]. However, as discussed before, the size-based categorization of urban/rural communities in the sample can produce misleading results, which should be interpreted with caution.

Consistent with the findings of Lakew and Haile (2015), we found significant variations in tobacco smoking across administrative regions [33]. In addition, we found significant geographic variations in the neighboring communities and between lowlanders and highlanders. There are a number of factors that could be attributed to the geographical variations in tobacco smoking in the administrative regions, the higher likelihood of smoking in neighboring SSN community clusters bordering Gambela and a higher likelihood of smoking in lowlanders. These factors include differences in regional availability and accessibility of tobacco products; differences in regional tobacco control policies and regulations; differences in demographics; and differences in religious and cultural practices. For example, Gambela is a lowland where people are culturally unique. Unlike the rest of Ethiopia, smoking is not a taboo for women in this region. Tigray, on the other hand, has a long-standing ban on consumption of khat, banned its cultivation in 2009 and prohibited smoking in public places in 2015. The lowest smoking prevalence rate in Tigray could partly be attributed to stringent regulatory measures, along with the social acceptance toward these measures, which together makes them fairly effective in deterring smoking.

Finally, the results on the time variable suggest that the probability of smoking decreased by 37% in the 2016 survey year, when compared to the 2011 survey year. It is our assertion that the lower reported incidence of smoking in 2016 should be viewed with some caution and not be interpreted as if the prevalence of smoking has declined over time. There are a number of reasons for our concern. First, each survey year consists of a randomly selected sample of households and it does not track the smoking behavior within a household. Second, the sample size is higher in the 2011 survey and the participation rate for males, who are six times more likely to smoke than females, is also higher at 43.7%, compared to 42.4% in the 2016 survey. Third, other data sources indicate a higher prevalence rate of smoking in Ethiopia than reflected in these samples, separately or combined.^17^ Fourth, although the Ethiopian parliament ratified the WHO framework on tobacco control in 2014 and eventually passed the anti-tobacco bill in 2015 prohibiting smoking in public places, it could be overreaching to attribute the results on time effects to these regulations. More specifically, while WHO framework prohibits tobacco advertising and promotion, sales of single cigarettes and sales to minors, and controlling of illicit trade of tobacco products, in Ethiopia all these banned practices continue to be widespread not only due to lack of awareness among users about their banning, but also the lack of any enforcement measures [46]. Similarly, smoking in public places was widely observed in the capital city.

### Model 2: interaction of region with khat

The result that khat chewers residing in the various regions differ considerably in their likelihood of smoking tobacco, provides some important insights regarding the local social osmosis of smoking behavior. For instance, individuals who live in the Eastern region and chew khat are almost 21 times more likely to smoke tobacco than Tigray residents. In addition, obtaining a statistically insignificant estimate for the main effect of the likelihood of smoking for Eastern residents (in reference to Tigray residents) when including the (Khat- regional) interaction term, suggests that the regional difference in smoking between the Eastern region and Tigray is primarily driven by the different prevalence of khat use in these regions.

Social osmosis is quite different in Gambela, the region with the highest incidence of smoking. As the results show, khat chewers in Gambela are only 56% more likely to smoke tobacco than Tigray residents. At the same time, with the inclusion of the regional interaction term, we see that the residents of Gambela are seven times (up from six times in Model 1) more likely to smoke than Tigray residents. This suggests that smoking behavior in Gambela is driven more by other factors such as those mentioned above in the discussion of the baseline results, rather than by khat chewing practices. Lastly, in Model 2, we obtained a slight decrease in the estimated likelihood of smoking by other religious groups compared to Islam (the reference category). One possible explanation is that percentage of those that smoke and chew khat is highest among Muslims at 57% (1146 out of 2009 respondents) and the overwhelming majority (86%) of Muslim khat chewers live in the Eastern region.

### Model 3: interaction of religion with khat

The main highlights of Model 3 results are as follows. First, overall, individuals from Orthodox and Protestant religious groups become less likely to smoke than the individuals who practice Islam, by 70 and 63%, respectively. Furthermore, the results for Catholics and Traditional religious groups become statistically insignificant. This qualitative change in the results, namely less (from more) likely to smoke than Muslim individuals, is in line with the descriptive statistics that show an overall higher prevalence of smoking among Muslim followers.

Second, despite the overall lower likelihood to smoke among Orthodox and Protestant individuals, those who chew khat among these groups are six times more likely to smoke tobacco compared to Muslim khat chewers. A possible explanation for these results is that smoking and khat chewing can be considered as “sinful” practices within the Orthodox and Protestant communities. However, those that engage in one sinful practice are considerably more likely to practice the other “sin” as they have already broken some moral expectations.

Third, we find that individuals working in agriculture are 35% more likely to smoke tobacco than the unemployed (the reference category), at 1% statistical significance. As mentioned earlier, it is a common practice in the rural areas to grow tobacco in home gardens for their own consumption, making the product easily available and accessible. While this could explain the results in the agriculture occupation in all models, it is not obvious as to why the statistically significant result is only obtained when including the religion-khat interaction variable in the analysis.

Finally, we turn attention to the results regarding two Oromia regional variables that are only statistically significant in Model 3. Specifically, Oromia residents are almost 128% more likely to smoke than Tigray residents. Once controlling for the khat chewing differences among various religious groups, the statistically significant result is in line with the descriptive statistics. The distance variable of Oromia community clusters from Harari also becomes significant at 10% confidence level and indicates that Oromia’s bordering communities with the Eastern region are 8% more likely to smoke than those located at farther distances from Harari. It seems that the cultural and religious similarities of these neighboring communities do matter for the prevalence of smoking.

### Implications for tobacco control policy

The findings of our study provide a number of policy implications for controlling tobacco consumption in Ethiopia. First, given the wide geographical variation in the prevalence of smoking, a “one size fits all” tobacco control policy that is national in scope and ignores geographical variation, may not be very effective. Instead, policies and regulations that take into account local and social contexts, would be more effective in reducing tobacco consumption.

One important consideration within local and social contexts is the prevailing practices of chewing khat. Currently, with the exception of Tigray, that has banned khat production since 2009 and also has a long-standing ban on consumption, other regions in Ethiopia have not regulated khat production or consumption at all. Since the use of khat can be considered a gateway to more tobacco smoking, an effective tobacco control policy could utilize the complementarity nature of these practices. Given that khat is a cash crop and one of the highest value export crops in the country, a production ban would affect the livelihood of many in the region. As a result, a production ban would be an unpopular policy that could drive the production into the unofficial economy. Its enforcement will be very costly and/or very lax and it is unrealistic to expect that it will be effective.

Instead, any measure that directly deters the consumption of khat could also lead to reducing tobacco smoking. Such policy can be particularly effective in regions with high incidence of both smoking and chewing khat, such as the Eastern region where khat users are 21 times more likely to smoke than Tigray residents who chew khat. In contrast, in a region like Gambela, where the incidence of smoking is the highest in the country but the khat chewing is not nearly as prevalent, measures that directly target smoking behavior could prove more effective than indirect policies for reducing khat consumption. Given the widespread culture of smoking, the high social acceptability of the practice, as well as the ease of accessing the locally grown tobacco in this region, regulatory measures based on price incentives such as taxation, or banning smoking in public spaces, may be difficult to change behavior in their own. Instead, to be more successful, they should be paired with educational campaigns about the adverse health and economic effects of tobacco smoking. Following the 2015 regulation, there was a lack of a nationwide awareness campaign on the dangers of smoking [46]. Hence there continues to be a need for awareness campaigns. These public campaigns can utilize various media sources such as radio, TV, cell text messages, or social media campaign where available. They may be run in partnership with community organizations that engage youth, agricultural development extensions activities, rural health posts, and be part of broader health education added to the school curriculum.

Our analysis also shows that although the use of khat is a more prevalent practice among Muslims than other religious groups, khat users among these latter groups are several times more likely to smoke tobacco than Muslim khat users. The implication of this finding is that policy measures (such as banning consumption) that deter the use of khat, may indirectly lead to deterring smoking behavior among Protestant and Orthodox individuals. Given that Muslims chew khat more as a customary practice,^18^ it could be more difficult to deter consumption of khat by an outright consumption ban. Instead, educational campaigns that raise awareness about the health hazard of both smoking and khat chewing may prove more effective. However, it is important that these educational campaigns avoid any perceived stigma toward a specific community, but instead are administered in partnership with religious community leaders.

Although educational campaigns about the adverse health risks of smoking are considered as soft touch policy tools, they provide broad benefits and could prove more effective in the long term, particularly when targeting youth. Youth with awareness about the health hazards of smoking are more likely to avoid starting smoking at a young age. One would expect that individuals, who have not engaged in smoking practices at a young age, are less likely to smoke later in life. A number of findings in our study provide support for a youth-targeted educational approach. For instance, since people are more likely to smoke if other household members smoke, educational efforts aggressively targeting youth could provide a resistance mechanism for imitating the harmful practice of smoking. Similarly, we find that older age groups are more likely to smoke and it is desirable to reduce the incidence of smoking within this group. It would be difficult, however, to deter smoking behavior using educational campaigns among those who have been engaging in the practice for a long time. Price incentives policy measures such as tobacco excise taxation may work more effectively for them instead of educational campaigns.

Other groups expected to respond more to tobacco tax/price increases are the urban dwellers and the professional workers who primarily use cigarettes. Currently, the tobacco tax rate in Ethiopia is 13.9%, while the WHO benchmark for tax rate is at 70% [6]. It appears that further increases in the tobacco excise tax rate in Ethiopia are feasible were Ethiopia attempting to be in line with the WHO benchmark.

Overall, the various policy examples above suggest a need for a multipronged policy approach in line with the WHO FCTC framework. These include taxation, smoking bans in public places, advertising and packaging requirements for tobacco products, along with educational campaigns targeting youth and other communities to raise awareness about the harmful health effects of tobacco smoking. In addition, undertaking educational campaigns that raise awareness about khat’s hazardous health effect should be considered and implemented first and foremost on the basis of their own merits in reducing the harmful effects of khat use with the potential complementarity of indirect effects in deterring smoking as an added benefit.

There are a few limitations of this study, which are primarily driven by the quality of data. First, the data on smoking any type of tobacco was self-reported and may be subject to recall errors. Additionally, social norms or stigma may prevent the young and women from reporting, thereby leading to an under-reporting of the rates [26]. Second, the DHS wealth index has been criticized for not being able to distinguish extremely poor households from poor households and being too focused on urban indicators in its construction [49]. Third, DHS are cross-sectional surveys and the pooled survey data used in this study do not allow us to track the smoking behavior of individuals over time. Finally, factors that affect the frequency of smoking may differ from those affecting its use, but these factors are not analyzed in our study.

## Conclusions

Using 2011 and 2016 Ethiopian Demographic and Health survey and a random intercept logistic model with two-stage residual inclusion estimation method, this study examines the influence of various individual, household, and community level factors on tobacco smoking in Ethiopia. The results show that chewing khat and geographic regions are statistically significant determinants of tobacco smoking even after controlling for various socioeconomic and demographic factors. Additional analysis including interactions between regions and khat use indicate wide inter-regional variations in tobacco smoking by khat users. Furthermore, examining the interaction between religion and khat use provides valuable insights about the prevalence of smoking among khat users of different religious groups.

Our findings suggest that a multipronged policy approach that combines various policy tools such as taxation, smoking bans in public places, educational campaigns, etc., could yield more effective results in tobacco control. To address the diverse population of Ethiopia, the implementation of these policy tools should account for regional variation, the local social contexts, as well as the complementary nature of smoking and khat chewing practices.

## Additional files

Additional file 1: Statistical Notations for the Methodology Section. (DOCX 20 kb)

Additional file 2: Baseline Model Results for 2011 and 2016 Survey Years. (DOCX 28 kb)

## Data Availability

This study used a dataset available from USAID’s The DHS Program and can be accessible at https://dhsprogram.com/data/dataset/Ethiopia_Standard-DHS_2016.cfm?flag=0

## Abbreviations

CSA: Central Statistical Agency
DHS: Demographic and Heath Surveys
WHO: World Health Organization

## Footnotes

1 See: https://www.tobaccocontrollaws.org/legislation/country/ethiopia/summary

2 See Economist magazine for more details at https://www.economist.com/middle-east-and-africa/2017/01/21/a-boom-in-qat-in-ethiopia-and-kenya.

3 It should be noted that we restricted our analysis to age group 15–49 as the DHS did not collect data for females aged 50–59.

4 Detailed information on survey methodology can be found elsewhere [see reference [34, 35].

5 These types of models have been previously employed using the DHS (See reference [51]). The details of the statistical notations are provided in the Additional file 1 for interested readers

6 We decided to exclude the use of smokeless tobacco such as chewing tobacco, snuff by mouth or nose etc. from the analysis since less than 1% of the respondents reported to use smokeless tobacco in combined 2011 and 2016 survey data.

7 The Eastern region comprises of Dire Dawa, Harari and Somali. Dire Dawa and Harari administrative regions are cities (although delineated as independent regions) adjacent to the Somali region. The dwellers of these cities share many social aspects with people residing in Somali region in terms of their religion, ethnic backgrounds and khat consumption practices.

8 DHS measures the cluster’s elevation/altitude (in meters) from the SRTM (Shuttle Radar Topography Mission) DEM (Digital Elevation Model) for the specified coordinate location. Elevations are regularly spaced at 30-arc sec or approximately 1 km. The altitude variable is converted into log values for our analysis. It should be noted that altitude information is missing for 47 community clusters (*N* = 1988). As a result, observations from these clusters were not included in the analysis.

9 As noted before the size-based categorization of rural/urban areas may provide some misleading results which should be taken with caution.

10 It should be noted that only 4% (*N* = 2453) of the total sample (*N* = 56,644) had missing information and was excluded from the analysis. Out of 2453 missing cases, 1988 observations were excluded for missing altitude information (see footnote 8) and only 23 observations had missing smoking information. Hence, the remaining missing data constitutes less than 1% of the total sample size.

11 We also ran the baseline model separately for 2011 and 2016 survey periods. We found no qualitative differences as most of the coefficients had the same sign and statistical significance in both survey periods. The results can be found in the additional file 2. In addition, we ran a test for the statistical difference of coefficients across the two surveys using a seemingly unrelated estimation method. The test results suggest that there is no statistically significant difference between the coefficients of the two regression equations, with the exception of age and occupation category. Therefore, we report the pooled cross-section analysis which includes a time dummy that captures time effects in tobacco smoking between the two survey years. As expected with the larger sample size, the pooled data yields higher precision of statistical estimates by reducing standard errors.

12 For instance, a considerably higher percentage of respondent that belong to Islam chewed khat (80% of 9464 responders who chewed khat) or used both khat and tobacco (76% of 2009 respondents who used both).

13 Testing for the equality of coefficients on 40–44 and 45–49 age groups suggest that they could be aggregated.

14 For more details see http://news.bbc.co.uk/2/hi/africa/2203489.stm and https://www.ethiopianreview.com/index/8178

15 The distance variable attempts to capture whether the social context similarities of the Oromia’s communities bordered with the Eastern region affect the likelihood of smoking. Harari is used as the focal point in the Eastern region since it has the second highest prevalence of smoking after Gambela.

16 In addition, the descriptive statistics from the two DHS surveys used in this study suggest that the lowest income groups (the bottom three quintiles) report a higher prevalence of smoking in 2016 than in 2011. This is concerning, since the elevated health risk from smoking among the poorest population can further affect their economic situations adversely, due to potential loss in income if they become ill and are unable to work.

17 According to the Global Adult Tobacco Survey (GATS) in 2016, there were around 3.4 million tobacco users and 1.9 million adult manufactured cigarette smokers in Ethiopia [48]. According to the World Bank’s World Development Indicators, Ethiopia’s population with ages 15–64 were estimated at 56.7 million in 2016 and 47.5 million in 2011. This indicates a prevalence rate of 6% for tobacco users and 3% for manufactured cigarette smoking.

18 See the Ethiopian media article http://addisstandard.com/commentary-khat-chat-ethiopia-criminalize-not/ to gain some further insights on khat use [50].
