# Supplementary material for: Factors affecting tobacco smoking in Ethiopia: evidence from the demographic and health surveys
Source: BMC Public Health. 2019 Jul 12;19:938. doi: 10.1186/s12889-019-7200-8 (PMC6624889; doi:10.1186/s12889-019-7200-8)
Supplement: Supplementary file 1 — Statistical Notations for the Methodology Section. (DOCX 20 kb) [file 12889_2019_7200_MOESM1_ESM.docx]

**Additional File -1: Statistical Notations for the Methodology Section**

Suppose the likelihood of smoking tobacco for i^th^ individual in the j^th^ community cluster is given by:

$logit\left( \frac{\Pr\left( Y_{ij}=1 \right|x_{ij},\zeta_{j})}{\Pr\left( Y_{ij}=0 \right|x_{ij},\zeta_{j})} \right)=\alpha+ \beta^{'} x_{ij}+ \zeta_{j}$ (1)

where α is the constant term, β is a vector of regression coefficients for each of the covariates $x_{ij}$ and ζj is a random intercept. The random intercept logistic model can be viewed as a latent-response model,

$Y_{ij}^{*}=\alpha+ \beta^{'} x_{ij}+ \zeta_{j}+ \varepsilon_{ij}$ (2)

where $Y_{ij}$ = 1 if $Y_{ij}^{*}$>0 and 0 otherwise, and $\varepsilon_{ij}$ is assumed to have a logistic distribution with mean 0 and variance π^2^/3. All omitted community-level covariates that cause some individuals to smoke tobacco are represented by the random intercept, which is assumed to be normally distributed with a zero mean and variance ψ. The degree of correlation between observed responses on two individuals i and i' from the same community cluster can be quantified in terms of the intra-cluster correlation (ρ) as:

$\rho=\frac{\psi}{\psi+ \frac{\pi^{2}}{3}}$ (3)

where $\psi$ is the cluster-level variance and $\psi+ \frac{\pi^{2}}{3} is$the total variance. The use of multilevel modelling also corrects standard errors of the estimated coefficients for intra-cluster correlation (heteroscedasticity).

In the two-stage residual inclusion estimation method, equation (2) in the second stage can be modeled as:

$Y_{ij}^{*}=\alpha+ \beta^{'} x_{ij}+ \zeta_{j}+\hat{e}_{ij}+ \varepsilon_{ij}$ (4)

where 𝑒̂_ij_ are the residuals from the first-step estimation.
