# Supplementary material for: Factors affecting tobacco smoking in Ethiopia: evidence from the demographic and health surveys
Source: BMC Public Health. 2019 Jul 12;19:938. doi: 10.1186/s12889-019-7200-8 (PMC6624889; doi:10.1186/s12889-019-7200-8)
Supplement: Supplementary file 2 — Baseline Model Results for 2011 and 2016 Survey Years. (DOCX 28 kb) [file 12889_2019_7200_MOESM2_ESM.docx]

| **Additional File -2: Baseline Model Results for 2011 and 2016 Survey Years** | | |  |  |
| --- | --- | --- | --- | --- |
|  | **2011** | **2016** | |  |
| **Variables** | **Odds ratio**  **(95% CI)** | **Odds ratio**  **(95% CI)** | |  |
| ***Fixed part*** |  |  | |  |
| ***Individual-level variables*** |  |  | |  |
| Chewing Khat in the 30 days preceding the survey | 8.140*  (6.608, 10.026) | 8.314*  (6.555, 10.545) | |  |
| Sex (Ref: female) | 8.044*  (6.127, 10.562) | 6.239*  (4.685, 8.307) | |  |
| Age (ref: 15-19) |  |  | |  |
| 20-24 | 2.974*  (2.102, 4.209) | 6.187*  (3.861, 9.914) | |  |
| 25-29 | 4.756*  (3.259, 6.943) | 9.381*  (5.829, 15.097) | |  |
| 30-34 | 6.425*  (4.292, 9.619) | 12.898*  (7.861, 21.163) | |  |
| 35-39 | 6.281*  (4.149, 9.509) | 15.542*  (9.311, 25.942) | |  |
| 40-49 | 7.331*  (4.912, 10.943) | 16.135*  (9.556, 27.246) | |  |
| Marital Status (ref: Not married) |  |  | |  |
| Married | 1.097  (0.864, 1.394) | 1.045  (0.821, 1.330) |  |  |
| Divorced | 2.008*  (1.475, 2.734) | 1.739*  (1.249, 2.423) |  |  |
| *1% significance level, ** 5% significance level, ***10% Significance level | | | | |

| **Additional File -2: Baseline Model Results for 2011 and 2016 Survey Years** | | |
| --- | --- | --- |
|  | **2011** | **2016** |
| **Variables** | **Odds ratio**  **(95% CI)** | **Odds ratio**  **(95% CI)** |
| Education (ref: No Education) | | |
| Primary | 0.972  (0.811, 1.165) | 1.044  (0.835, 1.306) |
| Secondary | 0.746**  (0.563, 0.990) | 1.142  (0.856, 1.524) |
| Higher | 0.515*  (0.360, 0.737) | 0.667**  (0.483, 0.921) |
| Occupation (ref: Unemployed) |  |  |
| Professional/Clerical/Sales/Skilled/Services | 1.442**  (1.094, 1.902) | 0.686**  (0.521, 0.905) |
| Agriculture | 1.403**  (1.071, 1.837) | 0.890  (0.661, 1.198) |
| Unskilled Manual/Other | 2.574*  (1.517, 4.367) | 1.074  (0.751, 1.536) |
| ***Household-level variables*** |  |  |
| Household wealth quintile (ref: Very Rich) |  |  |
| Quintile 1 (Very Poor) | 2.604*  (1.809, 3.747) | 2.473*  (1.635, 3.741) |
| Quintile 2 | 1.658*  (1.158, 2.374) | 2.051*  (1.336, 3.149) |
| Quintile 3 | 1.614**  (1.091, 2.387) | 2.081*  (1.342, 3.229) |
| Quintile 4 | 1.213  (0.865, 1.701) | 1.057  (0.675, 1.656) |
| Household members smoke inside the house | 13.609*  (11.082, 16.713) | 9.616*  (7.891, 11.718) |
| *1% significance level, ** 5% significance level, ***10% Significance level | | |

| **Additional File -2: Baseline Model Results for 2011 and 2016 Survey Years** | | |
| --- | --- | --- |
|  | **2011** | **2016** |
| **Variables** | **Odds ratio**  **(95% CI)** | **Odds ratio**  **(95% CI)** |
| Religion (ref: Islam) |  |  |
| Orthodox | 1.126  (0.861, 1.471) | 1.344**  (1.029, 1.755) |
| Catholic | 4.624*  (2.532, 8.444) | 1.286  (0.574, 2.880) |
| Protestant | 1.467***  (0.984, 2.187) | 1.894*  (1.301, 2.757) |
| Traditional/Other | 3.442*  (1.935, 6.122) | 4.176*  (2.232, 7.811) |
| ***Community-level variables*** |  |  |
| Place of residence (ref: Rural) | 2.509*  (1.686, 3.733) | 1.884*  (1.247, 2.846) |
| Administrative regions (ref: Tigray) |  |  |
| Addis Ababa | 3.040*  (1.397, 6.613) | 4.307*  (2.164, 8.572) |
| Affar | 1.868  (0.837, 4.167) | 1.777  (0.846, 3.730) |
| Amhara | 0.724  (0.340, 1.544) | 0.665  (0.305, 1.450) |
| Oromia | 1.137  (0.508, 2.541) | 2.080  (0.844, 5.126) |
| Benishangul-gumuz | 4.853*  (2.313, 10.182) | 4.991*  (2.573, 9.678) |
| SNNP | 4.688**  (1.217, 18.066) | 6.062*  (2.115, 17.381) |
| Eastern (Dire dawa, Harari and Somali) | 2.391*  (1.191, 4.798) | 2.248*  (1.181, 4.277) |
| Gambela | 10.494*  (4.181, 26.340) | 4.897*  (2.266, 10.583) |
| Distance of SNNP Region from Gambela | 0.713**  (0.515, 0.986) | 0.656*  (0.500,0.864) |
| Distance of Oromia Region from Harari | 1.065  (0.950, 1.192) | 0.878  (0.740, 1.040) |
| *1% significance level, ** 5% significance level, ***10% Significance level | | |

| **Additional File -2: Baseline Model Results for 2011 and 2016 Survey Years** | | |
| --- | --- | --- |
|  | **2011** | **2016** |
| **Variables** | **Odds ratio**  **(95% CI)** | **Odds ratio**  **(95% CI)** |
| Altitude distance from the sea level | 0.583*  (0.404, 0.841) | 0.585*  (0.430, 0.796) |
| β_ehat_ | 1.302  (0.900, 1.884) | 1.656**  (1.107, 2.477) |
| ***Random part*** |  |  |
| ρ^a^ | 0.18 | 0.14 |
| ψ^b^ | 0.718  (0.529, 0.974) | 0.539  (0.393, 0.740) |
| LR test statistic^c^ | 230.72* | 103.25* |
| Level 1 Units (N) | 27,838 | 26,353 |
| Level 2 Units | 571 | 622 |
| *1% significance level, ** 5% significance level, ***10% Significance level  ^a^ Intracluster correlation  ^b^ Variance of the random-intercept term  ^c^ Comparing random-intercept logistic model against ordinary logit model. | | |
